# Ras Multimers on the Membrane: Many Ways for a Heart-to-Heart Conversation

**DOI:** 10.3390/genes13020219

**Published:** 2022-01-25

**Authors:** E. Sila Ozdemir, Anna M. Koester, Xiaolin Nan

**Affiliations:** 1 Cancer Early Detection Advanced Research Center, Knight Cancer Institute, Oregon Health & Science University, 2720 S Moody Ave., Portland, OR 97201, USA; ozdemirs@ohsu.edu; 2 Program in Quantitative and Systems Biology, Department of Biomedical Engineering, Oregon Health & Science University, 2730 S Moody Ave., Portland, OR 97201, USA; koestera@ohsu.edu

**Keywords:** Ras, GTPase, dimers, multimers, nanoclusters, membrane proteins, nanodomains, spatial regulation

## Abstract

Formation of Ras multimers, including dimers and nanoclusters, has emerged as an exciting, new front of research in the ‘old’ field of Ras biomedicine. With significant advances made in the past few years, we are beginning to understand the structure of Ras multimers and, albeit preliminary, mechanisms that regulate their formation in vitro and in cells. Here we aim to synthesize the knowledge accrued thus far on Ras multimers, particularly the presence of multiple globular (G-) domain interfaces, and discuss how membrane nanodomain composition and structure would influence Ras multimer formation. We end with some general thoughts on the potential implications of Ras multimers in basic and translational biology.

## 1. Introduction

The Ras small GTPases are well established as key regulators of cell physiology [1,2]. Residing on the inner leaflet of the membrane, Ras functions as a molecular switch that receives signals from numerous extracellular inputs and relays them to an array of intracellular effectors. A wide variety of cell surface receptors, such as growth factor receptors [3], integrins [4], G-protein coupled receptors [5], and immune receptors [6,7] can signal to Ras. Canonical Ras effector pathways including Raf-Mitogen Activated Protein Kinase (MAPK) [8,9,10], phosphatidylinositol-3-kinase (PI3K)-Akt-mammalian target of rapamycin (mTOR) [11,12], Ral guanine nucleotide dissociation stimulator (RalGDS) [13,14], Phospholipase C (PLC) [15], and many others [16] have been well studied. Depending on the upstream input and the cellular context, activated Ras can recruit specific subsets of effectors and execute appropriate cellular programs accordingly. Through these effector pathways, Ras controls nearly all aspects of cell physiology: growth, proliferation, apoptosis, metabolism, motility, to name a few [1].

For the central roles of Ras in cell physiology, it is not surprising that deregulated Ras activities are common in human diseases. As much as one third of all human cancers are associated with mutationally activated Ras, and mutations in Ras and its regulators are also direct causes in Rasopathies and neuronal developmental disorders [17,18,19]. Four decades after *RAS* was identified as a proto-oncogene, however, our success in targeting mutant Ras has been limited. Except for the recent progress in targeting Ras G12C [20,21] and potentially G12D [22], direct inhibition of mutant Ras has proven difficult. Efforts to target downstream effector pathways, most notably Raf-MAPK [23,24] and PI3K-Akt [25], are often met with tumor resistance that can frequently be attributed to activation of alternative Ras pathways [24,26]. To overcome these issues and effectively manipulate mutant Ras in human diseases, it is imperative to further understand how Ras operates on the cell membrane and interacts with its diverse signaling partners.

The capacity of Ras to participate in broad cellular processes reflects its ability to interface with variable input sources and generate context-specific outputs with consistency. Such versatility and robustness require that Ras activities—both the competence and the specificity of signaling—be modulated over multiple facets. As such, Ras could adapt to different cellular environments and engage specific subsets of signaling partners to invoke the desired cellular processes accordingly to the biological context. A multitude of mechanisms have been identified to regulate Ras activities in cells. Of these, GTP-loading [27,28], post-translational modifications [29] and membrane localization [19,30] have been well established. More recently, formation of Ras multimers, including Ras dimers and nanoclusters, has emerged as a new mechanism of regulation [31,32,33]. These mechanisms pertain to distinct aspects of Ras biology, although they are not entirely independent of each other.

**Figure 1:**
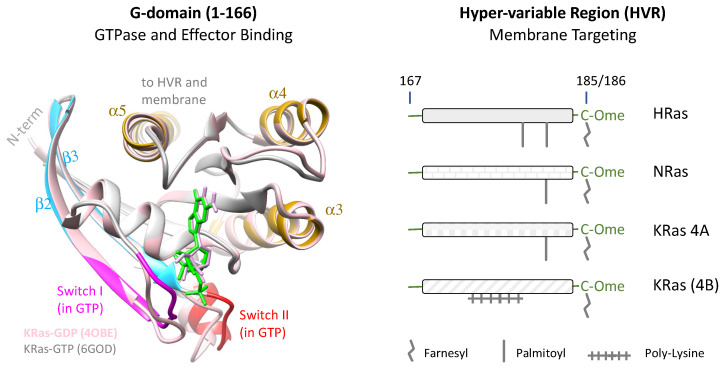
Ras domain structure. Left panel shows a ribbon representation of the heart-shaped Ras G-domain using overlaid KRas-GTP (Protein Data Bank (PDB) 6GOD; GTP shown in green) and KRas-GDP (PDB 4OBE; light pink; GDP in plum) structures as models. Several key elements most relevant to this review, including Switch I (residues 30–40, magenta), Switch II (residues 60–70, red), β-sheets 2 (β2, residues 37–46, sky blue), β3 (residues 49–58, sky blue), and α helices 3 (α3, residues 87–103, bronze), α4 (residues 127–137, bronze), and α5 (residues 152–164, bronze) are highlighted in the KRas-GTP model. Right panel depicts the hypervariable regions (HVRs) of the four major Ras isoforms, namely HRas, NRas, KRas (4A) and KRas (4B) along with the membrane targeting signals (farnesylation, palmitoylation, and the poly-lysine stretch). Figure adapted from ref [32].

**Figure 2:**
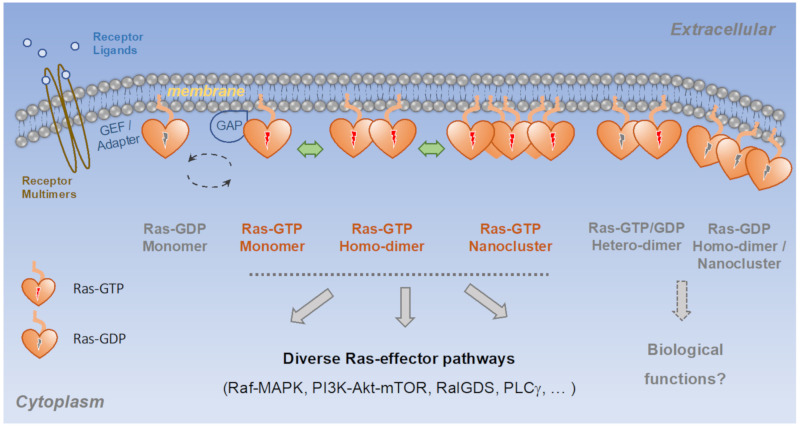
Ras dimers and nanoclusters in biological processes. Current evidence suggests that Ras exists as a mixture of monomers, dimers, and higher order multimers, where dimers and multimers could be homotypic, comprising either GTP- or GDP-bound Ras, or heterotypic, comprising both GTP- and GDP-bound Ras. At present, the physiological relevance of many of the Ras multimers remains unclear.

GTP-loading is a core mechanism for regulating all G-proteins, including the Ras small GTPases [27]. The first ~166 residues of Ras form a heart-shaped, G-domain that contains the GTPase and effector-binding motifs (Figure 1, left) [34,35]. Wild-type (WT) Ras cycles between an inactive, GDP-bound state and an active, GTP-bound state via counteracting enzymes including guanine exchange factors (GEFs) and GTPase-activating proteins (GAPs). Normally, Ras becomes GTP-loaded in response to upstream events that recruit Ras GEFs to the membrane, for example by Grb2 upon receptor tyrosine kinase activation [36,37]. GTP-loading causes conformational changes in the switch I and II regions that enable Ras to recruit cytosolic effectors (Figure 1, left) to the membrane for subsequent activation. The signaling process is terminated when the bound GTP is hydrolyzed into GDP, typically aided by GAPs since the intrinsic GTPase activity of Ras is low [35,38]. This scheme of regulation ensures that Ras-GTP is kept at low levels in resting cells and becomes elevated only upon proper upstream stimuli (Figure 2). Mutations in the *RAS* genes, commonly at the G12, G13, and Q61 residues, or loss of Ras GAPs such as NF1, impair GTP hydrolysis to result in upregulated Ras-GTP, ultimately leading to oncogenic transformation and other, pathologic conditions [17,18].

Membrane localization provides another critical layer of regulation for Ras activities in cells. Ras isoforms, notably the ubiquitous expressed H, N, and KRas (4A and 4B), are highly homologous in their G-domain sequences but differ substantially in their C-terminal 23–24 amino acids or so-called the HVRs (Figure 1, right) [39]. The HVR comprises a linker plus a membrane anchor, terminated with a CAAX (C=Cysteine, A=Aliphatic; X=Any) motif that is farnesylated post translation, followed by cleavage of the AAX [40,41]. At this stage membrane binding by the farnesylated cysteine is weak and requires strengthening by additional mechanisms [42,43]. NRas and KRas4A HVRs are further modified with one palmitoyl group whereas the HRas HVR takes two palmitoyl groups [44]. In contrast, KRas4B (hereafter KRas) contains a hexa-lysine polybasic sequence in its HVR that can electrostatically interact with acidic lipid headgroups to stabilize its membrane binding [45]. Aside from driving membrane localization of Ras, the modified HVRs are also responsible for the dynamic interactions of Ras with distinct membrane domains in an HVR-dependent (i.e., isoform-specific) manner [39,46]. As such, the Ras isoforms differ in effector specificity despite nearly identical G-domains and ability to bind to the same set of effectors in vitro [39,46,47]. Indeed, HRas, NRas, and KRas have overlapping but nonidentical functions [39,48,49,50] and distinct mutational spectra in human cancers [2,39,51,52]. Thus, while the Ras G-domain is functionally capable of mediating diverse biological activities, the signaling specificity may be primarily encoded by the HVR and its associated post-translational modifications [29].

The most recent and least understood mechanism is the formation of Ras multimers, including dimers and higher order oligomers (commonly referred to as Ras nanoclusters). Ras GTPases were traditionally considered monomeric G-proteins, but high-resolution imaging studies revealed that a significant fraction of Ras exists in multimers on the cell membrane and that formation of these structures is critical to the activation of Raf-MAPK and potentially other effector pathways [32,53]. When two Ras-GTP molecules come into contact, they could bring two effector molecules—each bound to a Ras-GTP monomer—into proximity, which may facilitate effector dimerization and activation. Indeed, at least one major Ras effector, the Raf kinase, depends on dimerization for activation [54]. Many members in the Ras signaling network such as MEK [55,56], extracellular signal-regulated kinase (ERK) [57,58], NF1 (a Ras GAP) [59,60], receptor tyrosine kinases [3], function as dimers. More broadly, dimerization and oligomerization have long been recognized as a general mechanism for regulating protein activities [61,62]. Ras-GDP could also dimerize and cluster [63]. In addition to Ras homodimers, heterotypic dimers formed between different types of Ras molecules, for example between Ras-GTP and Ras-GDP, have been proposed [33,64,65]. At present, the field is just starting to understand the makings and functions of the various Ras multimers (Figure 2).

This review aims to give an updated survey of existing literature on Ras dimers as a complement to several recent reviews on this topic [32,33,66,67,68,69]. We focus on Ras dimers since the same interactions are thought to mediate formation of Ras nanoclusters [70,71,72]. We begin with a summary of Ras dimer biology as it currently stands. Recent work has revealed some surprising features of Ras dimers, including the potential relevance of multiple dimer interfaces. We next discuss the various Ras dimer interfaces identified or proposed in recent works, followed by considerations on potential factors that regulate Ras dimer formation and function. Of these factors, we focus on anionic lipids, particularly phosphatidylinositol (4,5)-bisphosphate (PIP2) and phosphatidylserine (PS), which have been shown to interact with both the HVR and the G-domain of Ras on model membranes. In cells, the distributions of PIP2 and PS are heterogeneous, and both lipids interact with the cytoskeleton and participate in the dynamic remodeling of membrane topography. We conclude with a hypothetic model where versatile Ras signaling can be in part realized through Ras localizing to heterogeneous membrane nanostructures (‘nanodomains’), each featuring a unique combination of lipids and proteins and promoting specific types of Ras dimers to carry out specific subsets of Ras functions.

## 2. Ras Dimers as New Signaling Entities and Potential Therapeutic Targets

Until the last ~5 years, the dominant view of Ras GTPases had been that they are monomeric G-proteins, often compared with the family of trimeric G-proteins. This notion was affirmed with the observations that soluble Ras proteins containing only the G-domain are able to bind effectors in solution, and that multimers observed in crystals of Ras proteins seemed to be packing artefacts. The use of new imaging tools, including immuno-electron microscopy (EM) [73] and then super resolution microscopy [74,75,76,77], has allowed visualization of Ras proteins on the cell membrane with nanometer resolution. With these tools, it has been shown repeatedly that formation of Ras multimers on biological membranes is common. Immuno-EM imaging of prepared membrane sheets showed that ~40% of all Ras proteins exist in multimers, each consisting of 5–8 Ras molecules, and that the Ras multimers are spatially segregated depending on the isoform and nucleotide bound [78,79,80]. Super resolution imaging revealed that 10–20% of Ras molecules are present in dimers when expressed at near-endogenous levels, and the fraction of higher order multimers only becomes evident at higher expression levels [81].

Aside from nanoscopic imaging, Ras dimers have been probed with a multitude of other experimental approaches. In their original paper in 2000, Inouye et al. observed KRas-GTP dimerization on liposomes after exposure to a chemical crosslinker, which requires a lipidated HVR, and HRas G12V (GTP) dimerization in cells by means of β-galactosidase bimolecular complementation [82]. Attenuated total reflectance Fourier transform infrared (FTIR) spectroscopy and foster resonance energy transfer (FRET) [83] were used to detect NRas dimers on artificial membranes [84]. Single-molecule FRET was recently used to probe NRas dimers and measure the distance between specific sites on the two Ras protomers [85]. FRET and the related fluorescence life-time imaging microscopy (FLIM) [86] were used in many studies to detect proximities between Ras molecules in cells [65,67,84,87]. Negative-stain EM with structural fitting revealed an interesting, trimeric organization of KRas on a lipid monolayer [88]. Mass-spectrometry in combination with ultraviolet photodissociation was used to compare the dimerization tendencies of various G12X variants [89]. Nuclear Magnetic Resonance (NMR) has been a powerful tool to study transient and weak interactions [90] such as Ras dimerization in solution [91]. Most recently, NMR with paramagnetic relaxation enhancement (PRE) was used to study KRas dimerization on nanodiscs [71,92].

Importantly, several studies also reported the absence of Ras dimerization. On supported lipid bilayers, the Groves lab combined single-particle tracking, fluorescence correlation spectroscopy, and step-wise fluorescence bleaching to study potential homo-dimerization of HRas [93] and KRas [94]. Within a wide range of protein densities and lipid compositions, the authors did not find indications of dimer formation with either Ras isoform. The originally observed HRas dimers were later found to be artefacts due to laser-induced crosslinking of fluorophores [95]. Using a different set of approaches including time-domain fluorescence anisotropy and NMR, Kovrigina et al. also did not observe any G-domain interactions even when two HRas molecules were linked in tandem using flexible linkers of various lengths [96]. Rather than proving that Ras dimers do not exist, these studies suggest that biologically meaningful Ras-Ras interactions may only take place under certain conditions that, at present, are best afforded by biological membranes.

There has been increasing evidence in support of a critical role of Ras dimers in effector activation, at least in the case of Raf-MAPK signaling. Artificial dimerization of the KRas G12D mutant expressed at low levels (initially as membrane-bound monomers) induced strong Raf-MAPK activation and was sufficient to rescue cells from long-term serum starvation or Raf kinase inhibitor treatment [81]. Certain mutations at putative dimer interfaces (to be discussed in the next section) were shown to reduce or eliminate Raf-MAPK activation in cells [65,97]. In addition, small peptides such as the NS1 monobody [98], designed ankyrin repeat proteins (DARPins) [99], or a natural inhibitor of Ras named DIRAS family GTPase 3 (DiRAS3) (or ARHI) [100] that bind Ras at a putative dimer interface and presumably block Ras dimerization also inhibit Raf-MAPK and in some cases PI3K-Akt signaling [98]. Furthermore, Ras dimerization may mediate the formation of Ras nanoclusters, which were considered to be important for effector recruitment and activation [79,87].

The realization that Ras may function as dimers has profound implications in both basic and translational Ras biology. For an important signaling molecule like Ras, the use of dimers and higher multimers for signaling may yield performance advantages such as resistance to spontaneous fluctuations in Ras-GTP levels [101]. A number of studies have suggested a potential, tumor-suppressor role of WT Ras in mutant Ras-driven tumors [65,102,103,104,105,106]. However, WT Ras does not compete with mutant Ras for effector binding and should not interfere with oncogenic signaling of the latter. This may now be explained with a model where Ras signaling requires two Ras-GTP molecules, and therefore heterotypic Ras-GDP:Ras-GTP dimers are incapable of driving oncogenesis [33,64]. Consistent with this explanation, development of mutant Ras-driven tumors is often accompanied with a loss of heterozygosity [103,107,108]. In these cases, loss of the WT *RAS* allele and protein expression would eliminate nonfunctional Ras-GDP:Ras-GTP heterodimers and promote functional Ras-GTP:Ras-GTP homodimers, thereby enhancing mutant Ras-driven oncogenesis. As mutant Ras has been difficult to drug with conventional approaches, for example by restoring its GTPase activity or inhibiting its membrane localization, it may now be druggable by disrupting the dimers. On this front, aforementioned studies already demonstrated the potential utility of ‘Ras dimer inhibitors’ in targeting mutant Ras. The NS1 monobody, for example, binds both GTP- and GDP-loaded states of Ras and was shown to disrupt Ras dimerization, leading to decreased oncogenic signaling and transformation [98]. This effect was confirmed in vivo [109,110]. Interestingly, NS1 only inhibits KRas and HRas dimerization but not NRas due to a small difference between the isoforms in the putative dimer interface. The other peptides, DARPins [99] and DiRAS3 [100], may also be useful in therapeutics. While both GDP- and GTP-bound Ras seem capable of dimerization, some studies suggest that GTP-binding enhances dimerization [89,91]. If validated in vivo, this would lend a tool for selectively targeting GTP-bound Ras by inhibiting dimerization.

## 3. Ras Dimerization at Variable G-domain Interfaces

To better understand how Ras dimers form and function in cells, a critical step is to define the Ras-Ras dimer interface. Both experimental and computational approaches have been employed to identify the motifs and residues responsible for Ras dimer formation. On the computation side, homology or data-driven modeling [111,112] as well as template-based modeling [113] are widely used to generate physically fitting dimer structures, while molecular dynamics (MD) simulations extent the knowledge on the stability and interactions of the potential interfaces [91,114,115]. Recently machine learning approaches were coupled with the above to generate more data with increased accuracy [116]. On the experimental side, recent studies using FTIR, NMR, FRET, and EM in combination with point mutations and functional assays have been used to test and refine the predicted dimer interfaces [71,84,85,91,92,97,117].

Current efforts mostly focused on identifying Ras-Ras contacts at the G-domain [81,82]. A surprising finding from data thus far is the ability of Ras to dimerize at several different G-domain interfaces. The main three interfaces identified by several studies are as follows; two interfaces consist of α helices α4-α5 and α3-α4, and the last one uses the region encompassing β2-β3. We briefly describe these dimer interfaces below.

**Figure 3:**
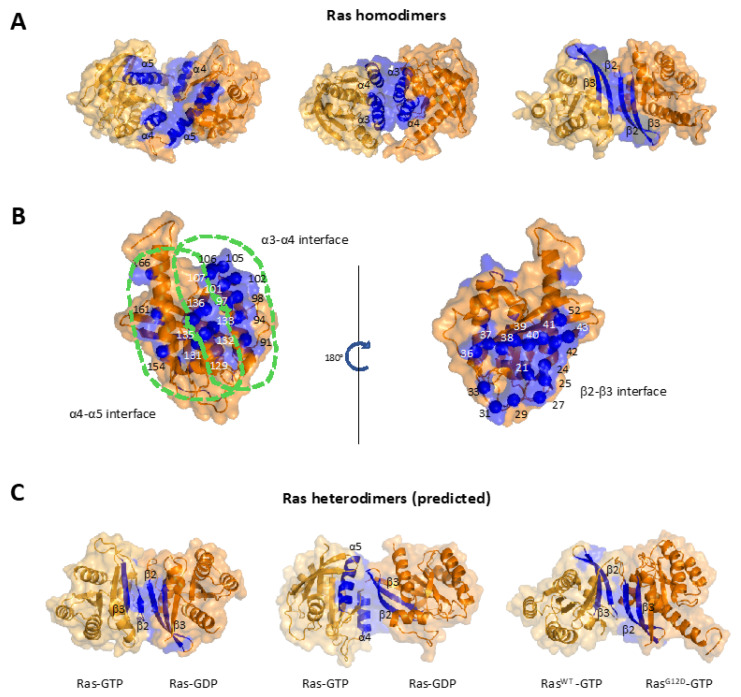
Various Ras G-domain dimer interfaces. (**A**) Three common interfaces identified by computational and experimental studies. These major interfaces can be classified as the α4-α5 interface (left panel), the α3-α4 interface (middle panel), and the β2-β3 interface (right panel). Blue regions indicate dimerization interfaces, two identical KRas monomers are colored with two shades of orange for the sake of visual clarity. (**B**) Mapping interface residues for all three dimer interfaces shown in (**A**) on the Ras G-domain, with the left showing those for the α4-α5 and α3-α4 interfaces and the right showing those for the β2-β3. (**C**) Predicted heterodimer interfaces. Left and middle panels are two predicted HRas-GTP/HRas-GDP heterodimer structures, featuring symmetric β-interface (left) and an asymmetric α-β interface (middle). On the right is a predicted structure for a KRas G12D-GTP/ KRas WT-GTP heterodimer. Predictions are made based on the methods described in Muratcioglu et al. [80].

The α4-α5 dimer interface mostly covers the α4 and α5 helices, the last two helices before the HVR. The helices correspond to G-domain residues 126–138 and 151–166, respectively. The detailed structural representation of this interface is shown in Figure 3A (left). The interface was originally proposed by Güldenhaupt et al. in the context of NRas dimers [84] and later identified computationally for KRas by Prakash et al. [117], Sarkar-Banerjee et al. [70], and Jang et al. [118]. This interface has now been experimentally tested for KRas [65,71,92] and NRas [85]. Ras-Ras interactions at this interface give rise to a dimer where the two promoters are ‘standing’ on their α4 and α5 helices relative to the membrane plane, leaving the effector region (switch I and II) well exposed. This dimer interface is therefore thought to be compatible with effector recruitment. This interface was targeted by the NS1 monobody [98], which blocks dimerization, signaling, and pancreatic tumorigenesis by mutant KRas [109,110].

The second interface is the α3-α4 dimer interface, which consists of another set of α-helices. It partially overlaps with the α4-α5 interface and roughly covers the G-domain residues 86–104 and 126–138. Muratcioglu et al. proposed this interface combining biophysical experimental techniques and structure prediction algorithms [91] (Figure 3A, middle). They further confirmed the predictions using dynamic light scattering, FRET and NMR experiments. Another study from the same group showed that charge reversal mutations in two critical residues on this interface decrease the dimerization [97]. Prakash et al. [117] and Sarkar-Banerjee et al. [70] also presented evidence for this interface.

The third Ras dimer interface involves two β sheets, namely β2 (residues 38–43) and β3 (residues 52–54), as well as part of the switch I (residues D33, I36, E37, and D38) (Figure 3A, right). This interface is more distinct than the previous two, both in its location on the Ras surface and in that it has some overlap with the switch I region, which is involved in effector binding. Muratcioglu et al. first proposed this interface in 2015 [91], where they named the corresponding dimer a ‘β-homodimer’ to distinguish it from the ‘α-homodimer’ (α3-α4). Although their computational predictions indicated that the β homodimer is more stable than the α homodimer, they commented that the relative populations and stability of the interfaces may change in full-length membrane anchored KRas. Later in 2015, Sayyed-Ahmad et al. characterized the β-sheet dimer interface for KRas using structure matching and all-atom MD simulations [119]. In the study, the authors suggested that, although formation of β-interface dimers is possible, it has a weak binding free-energy and it is unlikely to play significant roles in cellular KRas function.

The region constituting β2-loop-β3 was previously recognized as a ‘constitutive’ effector (or so-called ‘Ec’) region since mutations in many of the residues in this region, such as K42 and L53 (at this dimer interface) or I46, G48, and E49 (in the loop) were found to significantly impact Ras-mediated Raf activation with minimal effects on nucleotide binding, hydrolysis, or Raf binding [120]. Abankwa et al. named the combination of Ec and α5 a novel, switch III region. Some mutations in this region (such as D47A and E49A) occur in human cancers and augment HRas nanoclustering and signaling [121,122]. How these mutations alter Ras dimerization remains to be further elucidated, so do the functional roles of the β2-β3 interface.

These dimer interfaces may just be a subset of what may exist, as revealed in a recent collaborative, large-scale modeling effort [116]. With a Multiscale Machine-Learned Modeling Infrastructure (MuMMI), the authors ran an ensemble of over 100,000 simulations of active WT KRas on lipid bilayers with a variety of lipid compositions. Of these simulations, 10,939 showed Ras-Ras contacts. While the above dimerization interfaces were significantly populated in the simulations, a much broader range of Ras G-domain contacts was observed, suggesting that membrane-tethered Ras could dimerize at more G-domain interfaces than what we have experimentally captured. Even just considering the three dimer interfaces shown in Figure 3A, the residues involved in these dimer interfaces are distributed all around the G-domain (Figure 3B). In addition, Ras may use variations to the ‘typical’ dimer interfaces in certain scenarios, such as heterodimer formation (Figure 3C). Together, these data and analyses suggest that Ras dimerization may indeed be able to proceed with flexibility in the G-domain dimer interface.

Among its other ramifications, the presence of multiple dimer interfaces suggests a potential mechanism for Ras to assemble into nanoclusters. Barklis et al. reported trimers of fully processed, guanosine-5′-(βγ-imino)triphosphate (GppNHp)-bound KRas (farnesylated and carboxymethylated) on artificial membranes via negative staining EM. This prompted analysis of existing Ras structures in the PDB, which revealed many trimer arrangements in KRas, HRas, and NRas crystals [88]. Van et al. suggested that the trimers may use two G-domain interfaces, one partially overlapping with the α4-α5 interface and the other a novel interface involving α1 and switch I [66]. Sarkar-Banerjee showed that α3-α4 as well as α4-α5 interfaces play a role in the formation of larger oligomers by combining single molecule FRET and imaging experiments with computational modeling [70]. 

Formation of dimers and oligomers is a common feature shared by a large number of proteins, with the best known examples including the epidermal growth factor receptors (EGFRs) [123] and the Raf kinases [54,124,125]. What differentiates Ras dimers from the others is that Ras-Ras interactions appear to be relatively weak in general and that the transitions among the different dimer conformations may be readily achievable [70,116,117]. This unique feature may in part contribute to the functional versatility of Ras despite a much smaller functional domain compared with that of EGFR and Raf. The membrane environment in which Ras resides and functions is known to be highly heterogeneous with drastic, local variations in molecular composition and structure. This could be coupled with the intrinsic variability in Ras dimer conformations to achieve location-specific Ras functions.

## 4. Membrane Interactions Regulate Ras Enrichment, Orientation, and Dimerization

Ras proteins are now known to interact with membrane lipids via both the modified HVRs and their G-domains. Previous studies have established the roles of these interactions in the membrane binding of Ras and its enrichment in nanoscopic spatial domains in an isoform and nucleotide dependent manner. Recent work has started to suggest that these interactions also play a role in determining the orientation of Ras on the membrane with direct bearings on how Ras dimerizes.

**Figure 4:**
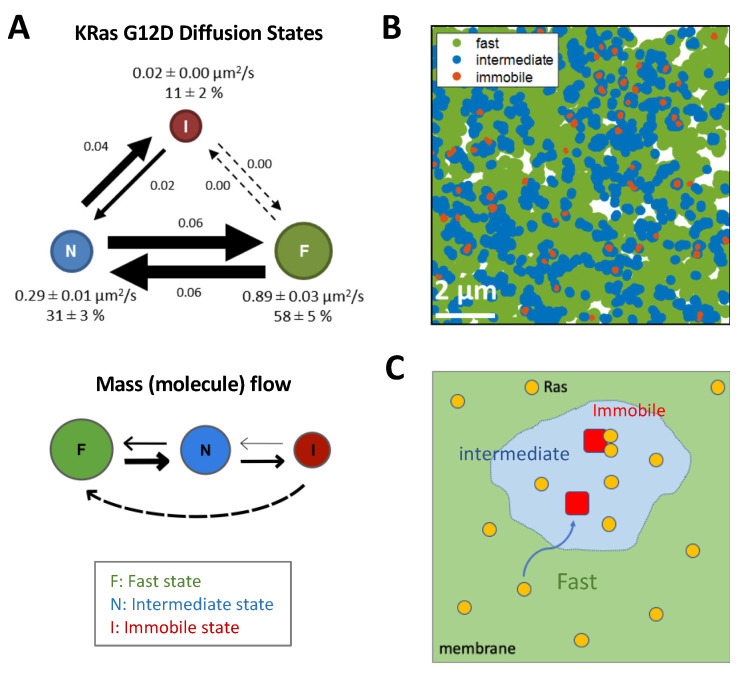
Ras diffusion on the membrane and localization to nanodomains. (**A**) KRas G12D molecules exhibit three diffusion states on the cell membrane, a fast (F) state, an intermediate (N) state, and an immobile (I) state, with state transitions between the F:N and N:I states but not F:I states. Analysis of this model suggests a net flow of Ras molecules from F to N to I states, and KRas is internalized while in the I state and recycled back to the membrane in regions that support the F state. (**B**) Mapping the locations of the three diffusion states revealed a nested organization of the membrane domains that confers each state, with each nanodomain that entraps Ras (immobile, red) living inside another, intermediate domain (blue), whereas most the membrane supports fast and free diffusion (green) of Ras. (**C**) Model depicting how Ras (yellow circles) diffuses on the membrane, frequently encountering the intermediate domains (light blue) and occasionally trapped in structures (red) within the latter, where they presumably form dimers and nanoclusters. (**A**,**B**) adapted from [130].

Most studies to date suggest a weak interaction between the Ras G-domains [89,91,92,115], therefore additional factors are likely needed to promote the formation of dimers and higher multimers. One such factor is to reduce the dimensionality of Ras diffusion, from a 3D solution phase to a 2D plane via membrane binding, and further, from a 2D plane to ~1D nanoscopic domains. This reduction in dimensionality is primarily achieved through interactions between the lipidated Ras HVR and the membrane with contributions from the G-domain [126,127]. Once on the membrane, the distinct HVRs and the associated lipidation patterns of each Ras isoform direct Ras to distinct, nanoscopic domains, leading to enrichment of Ras in highly confined spaces [78,79,80,128,129]. This targeting is achieved dynamically by Ras sampling the membrane (Figure 4) [130,131,132]. In the case of KRas, for example, the molecule diffuses on the membrane freely with fast mobility (diffusion coefficient or D ~1 µm^2^/s) until it enters domains that are ~200 nm in size and confer intermediate mobility (D ~ 0.3 µm^2^/s). Within the latter domain reside much smaller structures (~70 nm or smaller in diameter) that ultimately confine and immobilize KRas (D < 0.1 µm^2^/s), where KRas is internalized [130] and presumably forms dimers or nanoclusters during its residence [133]. 

Other Ras isoforms appear similar in their apparent diffusion and confinement behavior [134,135], although the underlying interactions are isoform-specific [132,136]. Computer simulations and cell biology studies suggest that the HVR-mediated spatial segregation of Ras is in large part driven by the interactions between its post-translational modifications, notably palmitoylation and prenylation, and membrane lipids. Whereas the fully saturated palmitoyl group prefers an ordered membrane domain such as the lipid rafts, the unsaturated farnesyl group (with ‘kinks’ in the aliphatic chain) prefers a less ordered domain [137]. The KRas HVR and similar polybasic peptides can bind to PS through both electrostatic interactions and specific molecular recognition [138,139]. 

Aside from the HVR, the Ras G-domain also makes contacts with the membrane and contributes to the spatial segregation and confinement, which could account for the spatial segregation of GTP- vs GDP-bound forms of Ras [140,141]. GDP-bound HRas and GTP-bound NRas incorporate cholesterol into their respective nanoclusters whereas GTP-bound HRas and GDP-bound NRas form cholesterol-independent nanoclusters [72]. Cholesterol was proposed to be important for de-mixing between different types of Ras nanoclusters [142]. KRas preferentially binds lipid bilayers containing PIP2 in part driven by its G-domain interactions with the anionic lipid [126,143,144]. Together, these mechanisms direct specific Ras forms (isoform and/or nucleotide-bound form) to distinctive, confined spaces where Ras-Ras interactions can be facilitated.

**Figure 5:**
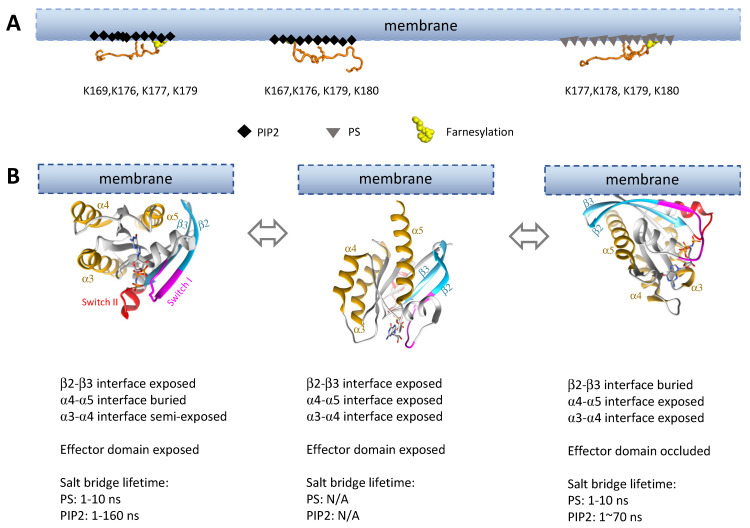
Membrane interactions dictate Ras orientations on the membrane, thus impacting accessibility of the dimer interfaces. (**A**) Interaction of KRas HVRs with different lipid moieties on the membrane. Two different HVR binding modes to the PIP2-enriched (black diamonds) membrane were observed, while one HVR binding mode was identified for PS-enriched (gray triangles) membrane [146,147]. Lysine residues playing an important role in those interactions are shown with stick representation, and the residue numbers are listed below the HVR structures. (**B**) Subset of possible conformations sampled by the G-domain on anionic (PIP2 or PS) membranes. All G-domain orientations were rendered to approximation based on literature [144,145,146,148,149,150].

Once in the nanodomains, the dimerization dynamics will largely be dictated by what is surrounding Ras. Dimerization requires the two Ras protomers to be properly oriented relative to each other, thus limiting the conformations compatible with dimer formation. Interactions between Ras and anionic lipids such as PS and PIP2 in its immediate environment, for example, may stabilize certain conformations, exposing some dimer interfaces and occluding others. The KRas HVR binds both PS and PIP2 with electrostatic interactions, which enables the HVR to adopt an orientation semi-parallel to the membrane (Figure 5A). The KRas HVR also specifically recognizes PS, and the presence of PS promotes the transition of the KRas HVR from a non-folded to a folded, helical conformation [138]. Since the KRas HVR is connected to the α5 helix, this may rigidify the linkage between KRas and the membrane and influence the overall orientation of KRas. The Ras G-domains also interact with anionic lipids in the membrane; depending on the set of residues involved, the overall orientation of Ras will be very different [141,145]. 

Experimental and computational studies show that KRas adopts many orientations on PS-containing membranes. The identified orientations vary somewhat among the studies. Two of the commonly observed orientations involve extensive membrane contacts of the Ras G-domain dimer interfaces: one at α4-α5 (Figure 5B, left) and the other β2-β3 (Figure 5B, right) [144,145,146,148,149,150]. The α3-α4 dimer interface could also be buried in the membrane (not depicted in Figure 5) [145]. In another well populated (perhaps dominant) set of orientations, KRas takes a membrane-distal conformation (with the α5 ‘standing’) in which all dimer interfaces are exposed (Figure 5B, middle) [148,150]. Each orientation exhibits a good degree of fluctuation, and Ras may transition between orientational states with relatively small kinetic barriers [148,150].

KRas binds to PIP2-containing membranes much more strongly than to PS-containing membranes despite the fact that the KRas HVR can specifically recognize PS [143,144,147]. Interactions between PIP2 and the KRas G-domain involve an overlapping set of basic residues as in the case of PS, giving rise to two similar KRas orientations on the membrane (Figure 5B). However, the salt bridges between these residues and PIP2 apparently last longer (some up to ~160 ns in simulations) than those with PS (up to ~10 ns) [144]. This likely means less flexibility in KRas orientations on PIP2-containing membranes and slower transitions between the different orientations. 

At present, it is unknown whether the membrane distal conformation remains a significant population on PIP2 membranes, or whether Ras dimerization differs significantly on PIP2- vs PS-containing membranes. It is clear, however, that anionic lipids have a major impact on Ras dimerization. Indeed, a recent NMR study reported that the presence of PS shifts KRas G12D dimers from symmetric α4-α5: α4-α5 dimers to asymmetric α4-α5: β2-β3 dimers [71]. This highlights the sensitivity of Ras dimerization to its surroundings and the importance of anionic lipids (among many other factors) in this process. 

GTP-binding and isoform differences also impact Ras-membrane interactions and likely dimerization. The conformational dynamics of KRas [72,151,152], HRas [72,140], and NRas [153,154] are all GTP-dependent (and different from each other). Among the major Ras isoforms, dimers of NRas appear to be the easiest to reconstitute *in vitro*, needing only a simple lipid bilayer comprising 1-palmitoyl-2-oleoyl-sn-glycero-3-phosphocholine (POPC) in multiple studies [84,85]. By contrast, in vitro reconstitution of HRas and KRas dimers needed addition of the Ras-binding domain (RBD) of Raf [115] or the use of nanodiscs (likely as a confinement mechanism) [92]. The origins of these isoform differences in ‘readiness to dimerize’ are yet to be determined, but a reasonable guess would be the differences in how they interact with the membrane.

## 5. Membrane Heterogeneity and Nanoscale Topography in Ras Dimerization

Most reconstituted membrane systems used to date for Ras studies are homogeneous lipid bilayers that lack some key features of actual biological membranes. These key attributes include the heterogeneous distribution of important lipid species (such as PS and PIP2), the exquisite physical structure of the local membrane, and the presence of potential scaffold mechanisms. Lateral segregation of proteins and lipids leads to transient or stable membrane domains common in biological membranes, to which other molecules can dynamically localize, creating a rich and complex environment for biological interactions [155,156]. We will discuss the potential impact of membrane heterogeneity and nanoscale topography on Ras dimerization in this section, and that of effectors and scaffold proteins next. 

The spatial distribution of PIP2 on the cell membrane is known to be heterogeneous [157,158]. PIP2, which makes up >90 % of all PIPs but comprises only ~1% of all membrane lipids, supports a wide-ranging spectrum of cellular functions by interacting with hundreds of proteins [159,160]. It is hypothesized that only a small fraction of PIP2 is freely available in the plasma membrane and that the majority is bound to, and released by, proteins in a dynamic manner [161,162]. The concentrated patches of PIP2 might in turn attract effector proteins resulting in activation of a variety of downstream pathways [158]. Indeed, new imaging techniques suggest that PIP2 is distributed as clusters and monomers. Using EM and freeze-fracture membrane preparations of live cells, PIP2 was shown to be highly concentrated at the rim of caveolae as well as in clathrin-coated pits [163]. Super resolution microscopy using fluorescently tagged anti-PIP2 antibodies also showed PIP2 clusters of 60–90 nm size in cells [162,164].

PS accounts for 10–20 % of all phospholipids in a typical cell and is found almost exclusively in the inner cytoplasmic leaflet of the membrane [165]. Using the Lact-C2 biosensor [166], evidence accumulated that a variety of intracellular proteins including KRas bind to or are influenced by PS in a charge-based manner to maintain their appropriate distribution within the cell [139,167,168]. Despite its abundance, PS is also distributed heterogeneously in the membrane. EM images of BHK cells transfected with Lact-C2-GFP showed that PS forms clusters at the inner leaflet of the membrane and that some, like PIP2, are associated with caveolae [169]. PS co-segregates with cholesterol, an important component of ordered membrane domains such as rafts, not only in the membrane but throughout subcellular compartments such as the early endosomes [169,170,171]. 

**Figure 6:**
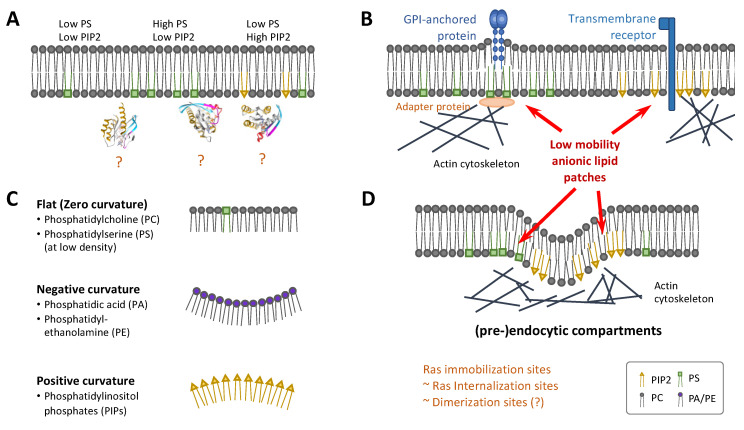
Membrane heterogeneity and topography in Ras dimerization. (**A**) Heterogeneous distribution of anionic lipids such as PS and PIP2 yields membrane regions with lipid compositions favoring some but not other Ras conformations, which could directly impact Ras dimerization. (**B**) Example mechanisms that couple actin polymerization with the local enrichment of PS (left) or PIP2 (right), yielding membrane patches of anionic lipids with relatively low mobility, which could entrap membrane proteins such as Ras. (**C**) Local enrichment of lipids could induce membrane curvature, potentially impacting Ras dimerization. (**D**) Endocytic and pre-endocytic compartments are examples of enriched anionic lipids coupled with actin polymerization and membrane curvature. There is evidence that Ras immobilization occurs in the same membrane nanodomains that internalize Ras, making some (pre-)endocytic compartments candidate sites for Ras dimerization and clustering. Parts of the figure were based on refs [155,174,175].

The spatial distribution of lipids is directly relevant to Ras dimerization and clustering. A recent immuno-EM study showed that different Ras nanoclusters have distinct lipid compositions: PIP2 was found to be enriched in GDP-bound HRas nanoclusters, all HRas and KRas nanoclusters contained PS and PI(3,4,5)P (PIP3). Another common anionic lipid, phosphatidic acid (PA), was found to be enriched in GTP-bound KRas nanoclusters [172]. Depletion of PS showed that clustering of GTP-bound KRas nanoclusters linearly depends on PS membrane content and that PS is vital for nanocluster formation and stabilization [172]. Depletion of PIP2 displaces KRas, but not HRas, from the plasma membrane to the Golgi, and in so doing diminishes KRas signaling [173]. As discussed earlier, the heterogeneous distribution of PS, PIP2, and other anionic lipids (such as PA), for example their clustering within or exclusion from the various Ras-associated nanodomains, will directly impact the dimerization properties of the Ras molecules residing in these domains (Figure 6A). However, the effect of these lipids on Ras dimerization is likely more than what was discussed in the last section.

Both PS and PIP2 have been involved in actin dynamics, which has been implicated in the spatiotemporal organization of Ras. Actin and its binding proteins form a dynamic network inside the cell that undergoes continuous remodeling. PIP2 is important for the regulation of the actin cytoskeleton by serving as a platform for protein recruitment, and changes in PIP2 concentration or clustering have a significant impact on actin dynamics. An increase in PIP2 leads to actin filament assembly [176,177] whereas a reduction in PIP2 results in actin cytoskeleton defects [178]. PS also directly interacts with actin-binding proteins such as talin and spectrin [179,180]. Hotspots of actin dynamics, some referred to as ‘actin asters’, have been implicated in the organization of membrane nanoclusters of Glycosylphosphatidylinositol-anchored proteins (GPI-APs) [181,182]. The long, saturated acyl-chains of PS likely serve as the molecular link between lipid-tethered GPI-AP clusters and the actin cytoskeleton [183]. These mechanisms yield patches of anionic lipids with the ability to trap other membrane proteins including Ras (Figure 6B).

An additional aspect of the local enrichment of these anionic lipids is their connection with membrane curvature, which has also been shown to influence the activity of many signaling molecules [184]. Biological membranes are not flat but covered in pits, protrusions, ruffles, and other types of structures. The generation and maintenance of these structures are fundamental to cell morphology and numerous other functions [185]. PIP2 directly interacts with Bin-Amphiphysin-Rvs (BAR) domain proteins to induce membrane curvature [186], and membrane curvature can be induced by the proximity of anionic phospholipids alone (Figure 6C) [157,187,188]. It was hypothesized that cholesterol associates with PS in membrane nanodomains and sufficiently separates the headgroups of PS to limit spontaneous curvature [187]. PS also plays an important role in the formation and stability of caveolae [189,190], which may indirectly regulate Ras nanoclustering [142].

A few recent studies investigated the relationship between the spatiotemporal organization of Ras proteins and membrane topography [191,192,193]. One study investigated the recruitment of the fluorescently labelled minimal membrane anchor of the NRas (tN-Ras) isoform from solution to fluorescently labelled liposomes of different diameters [191], suggesting that membrane curvature is a novel modulator of NRas lipid anchor and palmitoyl chain partitioning. A certain synergy may exist between bilayer lipid shape and membrane curvature in regulation of the recruitment of lipidated proteins to the membrane in general [192]. By growing cells on substrates with artificially engineered nanobars to create membrane curvatures, another recent study revealed that tH-Ras was concentrated at the curved ends of positively curved membranes compared to mutant KRas [193]. Immuno-EM confirmed enrichment and elevated nanocluster formation of tH-Ras in membrane regions with high curvature, whereas KRas and tK-Ras showed the opposite. The same study showed enrichment and elevated nanoclustering of PIP2 with increasing membrane curvature and the opposite for PS [193]. Even though Ras itself lacks structural features to detect membrane curvature, curvature modulations result in changes in lateral segregation of membrane lipids which can be sensed by Ras with its ability to sort distinct lipid head groups and acyl chains. Computer simulations suggested that membrane clustering of tH-Ras may also induce membrane curvature [128]. Reorientation of Ras on the membrane in response to membrane curvature could lead to either the occlusion or free accessibility of the dimerization interfaces and effector domains (Figure 6C). This could explain differences in downstream signaling of the different Ras isoforms depending on their surrounding membrane topography [67].

A unique combination of lipid-protein composition and the associated membrane topography defines a specific type of membrane nanostructure (which is generally and casually called a ‘nanodomain’). Among the potential nanodomains that Ras could be associated with and dimerize within, endocytic compartments represent an interesting class. Endocytosis, an indispensable process in eukaryotic cells, is driven by the interplay of local lipid and protein enrichment, membrane curvature and actin dynamics [194]. Clathrin-dependent endocytosis, for example, takes place in membrane regions enriched in PIP2 clusters that encourage membrane curvature [195]. The clathrin plaques and pits are further stabilized by actin filaments. Adaptor proteins are recruited to stabilized clathrin-lattices resulting in an additional increase in local PIP2 concentration creating a distinct membrane environment [194]. Incidentally, at least in the case of KRas, the membrane diffusion of Ras is coupled with endocytosis, where internalization of Ras likely occurs in membrane nanodomains that immobilize Ras (see also Figure 4) [130]. These nanodomains were previously hypothesized to be sites where Ras forms nanoclusters and recruits effectors [87,133]. As such, it is not surprising that the signaling activity of some Ras isoforms depends on endocytosis [196]. At present, however, it is unclear which type of endocytic compartments are conducive to Ras dimerization. Defining these and other types of Ras-associated nanodomains will be an important step to understanding Ras dimerization in cells.

## 6. Scaffold and Effector Proteins in Ras Dimerization

In addition to the structural elements within membrane nanodomains, extrinsic factors such as Ras-binding proteins may also regulate how Ras dimers are constructed and function. Since Ras exerts its biological activities via interactions with effectors and other signaling partners, understanding Ras dimers in complex with signaling partners and scaffold proteins is as important as (if not more than) understanding how Ras dimerizes on its own. To date, there has not been a detailed structure of full-length and fully modified Ras in complex with full-length effectors such as Raf or PI3K on a membrane. Nevertheless, exciting advances have been made in solving the structure of Ras in complex with the Raf RBD or both the Raf RBD and the cysteine-rich domain (CRD).

**Figure 7:**
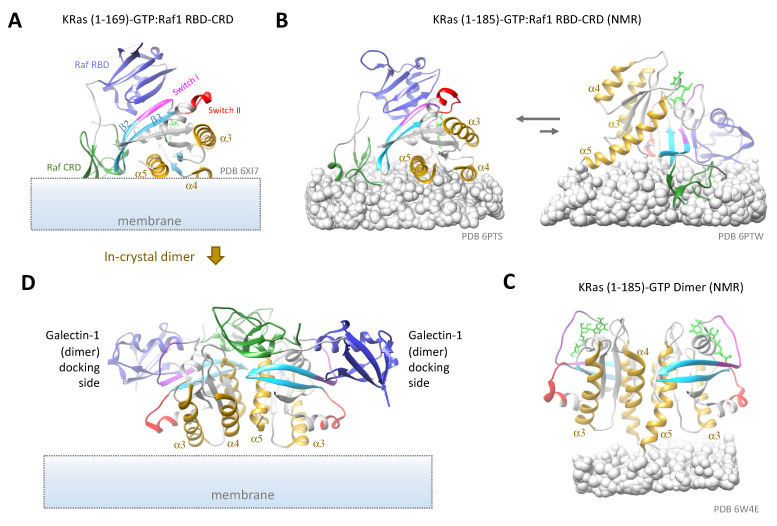
Ras conformations in complexes with binding partners. (**A**) Crystal structure of GTP-bound KRas G-domain in complex with Raf1 RBD-CRD, showing its orientations in approximation with membrane modeled in, as in ref [197]. (**B**) NMR structures of full length KRas-GTP in complex with Raf1 RBD-CRD, showing two different conformations of the complex relative to the nanodisc membrane (rendered as gray balls), with the left conformation being dominant at low complex concentrations. (**C**) NMR structure of full length KRas-GTP dimer on nanodiscs. (**D**) Potential dimer configuration present in the crystal structure of (A) (PDB 6XI7), as proposed by Cookis et al. [200]. The two Raf1 RBDs (blue) could serve as docking sites for Galectin-1 dimers, leading to formation of Ras nanoclusters [115]. The different proteins domains and motifs are labeled the same way as in (**A**). The HVRs in (**B**,**C**) are either partially buried in or visually blocked by the membrane.

Using X-ray crystallography, Tran et al. solved the structure of KRas G-domain complexed with a peptide comprising both the RBD and CRD of Raf1. The structure revealed extensive interactions of KRas with both the RBD and the CRD, with the CRD close to the β2-loop-β3 and the α5 helix of KRas. Aided by NMR spectroscopy, the authors modeled how this complex interacts with the membrane and proposed a model where the α4 and α5 helices of KRas as well as the CRD of Raf1 make close contacts with the membrane [197]. By fitting the structure to the possible KRas conformations observed in large-scale MD simulations [145], a membrane:KRas:Raf1 RBD-CRD model was inferred (Figure 7A). In this model, KRas adopts a conformation with its α4-α5 region juxtaposed to the membrane, similar to that shown in Figure 5B (left). This conformation enables good contacts between the CRD and the membrane but precludes dimerization at the α4-α5 (membrane occlusion) or the β2-β3 (blocked by the Raf1 CRD) interface. It also resembles one of the two observed conformations of KRas:Raf1 RBD-CRD on nanodiscs using PRE-NMR by Fang et al. (Figure 7B, left) [198], although the folding of Raf1 CRD differs significantly between the two. Dimerization via the α3-α4 dimer interface might still be possible, as suggested in a simulation study by Jang et al. [199]. In the PRE-NMR study, another, less populated conformation might also be compatible with Ras dimerization at both the α4-α5 and the α3-α4 interfaces (Figure 7B, right). Indeed, at increased KRas and Raf1 RBD-CRD concentrations, the equilibrium seems to shift towards the second conformation [198]. All these KRas conformations, however, deviate significantly from that seen in the KRas dimers observed on nanodiscs with PRE-NMR (see Figure 7C) [92].

In another study, Packer et al. demonstrated that the presence of Raf RBD induces robust dimerization of both KRas-GTP and HRas-GTP on artificial membranes containing 20% PS, where Ras dimerization likely takes place at the α4-α5 interface. Here, binding of Raf RBD causes an allosteric effect extending from the switch region (Ras-RBD binding site) to the α4-α5 dimer interface, which may have helped stabilize the α4-α5 exposed conformation to promote formation of the corresponding dimer [115]. Cookis et al. obtained the crystal structure of HRas (G-domain)/Raf RBD-CRD, which is nearly identical to that of KRas (G-domain)/Raf RBD-CRD [197], and proposed an alternative model for the tertiary Ras-Ras/Raf-Raf complex based on a α4-α5 Ras dimer. In this model, the Raf CRD is located between the two Ras protomers and kept away from the membrane [200]. Interestingly, the authors pointed out that this dimer model is present in the crystal (between two-unit cells) from which the KRas-GTP:Raf1 RBD-CRD structure was obtained [197] (Figure 7D).

**Figure 8:**
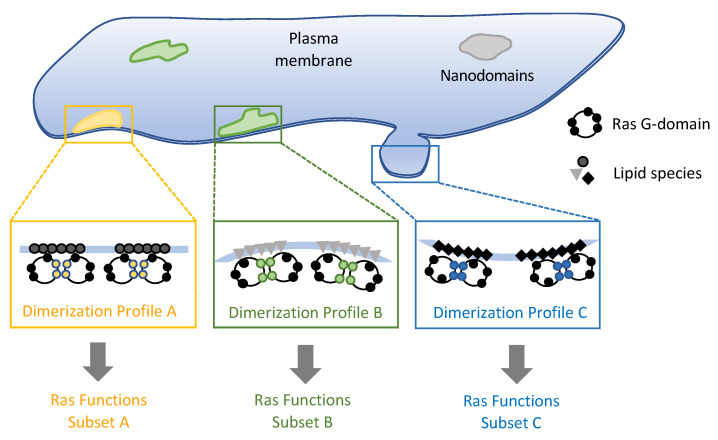
Model for nanodomain specific Ras dimer formation and signaling. Different Ras-associated membrane nanodomains feature unique lipid and protein compositions, physical structure, and associations with the cytoskeleton, among other properties. This leads to nanodomain-specific Ras dimerization profiles corresponding to different sets of biological functions.

While the discrepancies among these studies potentially arose from the different experimental and computational setups, the results thus far demonstrate the impact of binding partners on Ras conformation and dimerization on the membrane. The Raf1 CRD binds anionic lipids such as PS and PA and enhances Raf binding to Ras [201,202,203]. Compared with the conformations of Ras alone on the membrane [145], the additional interaction between Raf1 with anionic lipids seem to alter the conformational dynamics significantly (Figure 5 and Figure 8). The Buck and Nussinov groups have used computational simulations to show that monomers of Ras-Raf RBD-CRD complexes sample multiple conformations on PS-containing membranes, with the Raf CRD alternating between membrane proximal and distal configurations [203,204]. Both studies suggest the presence of conformations where two or more of the three Ras dimer helices (α3, α4, and α5) are well exposed, although these dimerization compatible conformations were not observed in the PRE-NMR study [198].

Another interesting insight from the studies discussed above is the role of Raf in stabilizing Ras dimers. Raf is known to dimerize through its kinase domain after binding to Ras [54,205] and in so doing may stabilize Ras-GTP multimers [206]. In the Packer et al. work, adding Raf1 RBD to HRas or KRas-GTP enabled robust dimerization on supported lipid bilayers and to a lesser extent, in solution [115]. Thus, the stabilization effect may be mediated via both the RBD (initial binding) and the Raf kinase domain (upon Raf dimerization, which follows Ras dimerization). As will be discussed below, this enhancement may synergize with other mechanisms such as scaffold mechanisms to ensure robust dimerization of the inherently weak Ras dimer where needed. In this regard, it will be interesting to see whether other Ras effectors can do the same through similar mechanisms. Of note, while bearing sequence homologies, the RBDs of Raf1, PI3K, and RalGDS, bind Ras with somewhat different orientations and contacts [207,208,209,210].

Scaffold proteins could further alter how Ras forms dimers, alone or in complex with signaling partners. Based on the high-resolution structures, Packer et al. also proposed that Galectin dimers, such as those of Galectin-1, can connect proximal Ras-RBD dimers into even larger and more stable signaling complexes to give rise to membrane condensates, which were implicated in Ras activation [211]. This provides an alternative model for Ras assembly into large multimers [115]. Indeed, Galectin-1 exists as dimers in the cytosol and has been shown to bind Raf RBD and stabilize HRas nanoclusters [212]. Another recent study provided an alternative mechanism for making large KRas-Raf signaling clusters (‘signalosomes’), where Galectin-3 served as a scaffold [213]. Galectin-3, which exists in solution as a mixture of monomers and pentamers [214], was previously implicated in augmenting KRas-GTP nanocluster formation and signaling [215,216]. Another lectin, Galectin-8, a dimer in solution, has also been implicated in KRas signaling [217]. Nucleophosmin and nucleolin may also stabilize KRas clustering by acting as scaffold proteins [218]. Additional scaffold proteins or mechanisms are yet to be identified. At present, it is unclear whether the role of these scaffold proteins is simply to make larger Ras (or Ras-effector) clusters or to alter the dimer conformation, or both.

## 7. Concluding Remarks

Since our last review on this topic [32], significant advances have been made in our understanding of Ras dimers. Ras dimer formation has now been recognized as a key step in the activation of Raf [65,81,98,219] and potentially other effectors [33]. In the pursuit of a better understanding of Ras dimer biology, the field has also gained rich, new insights into how Ras interacts with the membrane and its signaling partners. These new insights have contributed to the reviving enthusiasm toward drugging mutant Ras. Strategies that target the Ras dimer interface have shown their initial promises as novel therapeutics against mutant Ras, adding to the small but growing list of options for tackling this difficult drug target.

An important observation from existing work is that Ras can potentially dimerize at a variety of G-domain interfaces. The most notable is the α4-α5 dimer interface, which has been repeatedly observed in computational and experimental studies [85,92,117,118]. Also observed experimentally are the α3-α4 and β2-β3 interfaces (and the close variations), and their physiological relevance has yet to be further studied. In addition to dimerization using symmetric interfaces, asymmetric dimers using an α-β interface is also permitted and may facilitate transition from dimers to nanoclusters [71]. Heterotypic dimers between Ras-GDP and Ras-GTP are also likely and could account for the tumor suppressor effect of WT Ras [65,91]. While dimerization is common in signaling proteins, such a degree of variability in the dimerization interface could make Ras a unique case.

The possibility of using multiple dimerization interfaces implies that Ras dimerization is not a simple ‘on-off’ switch. Instead, by adjusting the ratios of the different dimer variants—including nanoclusters—the population and lifetime of the ‘competent’ dimers can be finetuned. The means to achieve this tuning can be manifold: local concentration of Ras, the surrounding lipid composition, the local membrane topography, and the presence of binding partners, among others. Biological membranes feature numerous types of nanoscale to microscale domains, each of which comprise a unique molecular composition, structure, and accessibility, and are thus ideal platforms to build a signaling module like one around Ras. Each type of membrane domain will therefore be associated with a unique ‘dimer’ profile, which in turn correspond to a specific subset of biological processes (Figure 8). Studies on Ras dimers, nanoclusters in cells as well as in reconstituted membrane systems have gained much insight into Ras-membrane interactions. Defining the specific types of Ras-associated membrane nanodomains and how they regulate Ras dimerization will be a critical next step toward understanding how Ras operates on the cell membrane.

While current work is mostly at a proof-of-concept stage, development of agents that manipulate Ras dimers as mutant Ras-targeted therapeutics is an exciting prospect for the field. Initial work assuming a single, well-defined dimer interface aimed to develop agents that block the dimer interface. At least one of such agents, the NS1 monobody, has demonstrated its utility in model systems [98,109]. The realization that Ras may use multiple dimer interfaces presents another opportunity for manipulating Ras activity—inducing the unproductive dimers, as demonstrated using the small molecule BI-2852 [71,220,221]. Aside from potential use as therapeutics, many of these agents will be useful tool molecules for Ras dimer research. 

In the past several there has been great success in combining experimental investigations with computer modeling in tackling the mysteries of Ras dimers. Results from multiple disciplines are reaching a critical mass and already starting to converge on some key points. There is a good reason to believe that a more exciting era for Ras biology is yet to come.

## Data Availability

Not applicable.
